# Changes in biodistribution on ^68^Ga-DOTA-Octreotate PET/CT after long acting somatostatin analogue therapy in neuroendocrine tumour patients may result in pseudoprogression

**DOI:** 10.1186/s40644-018-0136-x

**Published:** 2018-01-24

**Authors:** Martin H. Cherk, Grace Kong, Rodney J. Hicks, Michael S. Hofman

**Affiliations:** 1 Centre for Cancer Imaging, Peter MacCallum Cancer Centre, 305 Grattan Street, Melbourne, VIC 3000 Australia; 2 Department of Medicine / Sir Peter MacCallum Department of Oncology, University of Melbourne, Melbourne, Australia; 3 Department of Medicine, Monash University, Melbourne, Australia; 4 Department of Nuclear Medicine, The Alfred, 55 Commercial Rd, Prahan, VIC 3181 Australia

**Keywords:** Somatostatin, Octreotate, Ga-68, Neuroendocrine tumour, Positron emission tomography, DOTATATE, PET/CT, Octreotide

## Abstract

**Background:**

To evaluate the effects of long-acting somatostatin analogue (SSA) therapy on ^68^Ga-DOTA-octreotate (GaTate) uptake at physiological and metastatic sites in neuroendocrine tumour (NET) patients.

**Methods:**

Twenty-one patients who underwent GaTate PET/CT before and after commencement of SSA therapy were reviewed. Maximum standardized uptake values (SUVmax) were measured in normal organs. Changes in uptake of 49 metastatic lesions in 12 patients with stable disease were also compared. Serum chromogranin-A (CgA) levels were available for correlation between scans in 17/21 patients.

**Results:**

Mean thyroid, spleen and liver SUVmax decreased significantly following SSA therapy from a baseline of 5.9 to 3.5, 30.3 to 23.1 and 10.3 to 8.0, respectively (*p* = < 0.0001 for all). Pituitary SUVmax increased from 10.2 to 11.0 (*p* = 0.004) whereas adrenal and salivary gland SUVmax did not change. Tumour SUVmax increased in 7 of 12 patients with stable disease; CgA was stable or decreasing in 5 of these patients. 30/49 (61%) metastatic lesions had an increase in SUVmax and lesion-to-liver uptake ratio increased in 40/49 (82%) following SSA therapy.

**Conclusion:**

Long-acting SSA therapy decreases GaTate uptake in the thyroid, spleen and liver but in most cases increases intensity of uptake within metastases. This has significant implications for interpretation of GaTate PET/CT following commencement of therapy as increased intensity alone may not represent true progression. Our findings also suggest pre-dosing with SSA prior to PRRT may enable higher doses to be delivered to tumour whilst decreasing dose to normal tissues.

**Electronic supplementary material:**

The online version of this article (10.1186/s40644-018-0136-x) contains supplementary material, which is available to authorized users.

## Background

Neuroendocrine tumours (NETs) are a heterogeneous group of tumours, which arise most commonly in the gastreoenteropancreatic tract but can arise from any organ where neuroendocrine cells reside [1, 2]. These tumours have several biological properties in common including the presence of somatostatin receptor (SSTR) expression in the majority of tumours [3]. Five SSTRs have been characterized to date with SSTR-2 and SSTR-5 expression exhibited in 70–90% of all NETs [4].

The high prevalence of SSTR overexpression in NETs has enabled the use of synthetic somatostatin analogues (SSA) to control symptoms related to over production of biologically active amines and peptide hormones frequently associated with NETs and to possibly delay disease progression [5–8]. These are generally administered as slow-release formulations to increase patient convenience. Available long-acting SSA (LA-SSA) currently include octreotide (Sandostatin-LAR, Novartis, Switzerland) and lanreotide (Somatuline, Ipsen, France).

^68^Ga-DOTA-Octreotate (GaTate) PET, which binds to SSTR-2, is becoming increasingly available as a superior diagnostic technique to stage and restage patients with NET. It is also used to determine suitability for peptide receptor radionuclide therapy (PRRT) based on the degree of radiotracer uptake in the tumour [9]. PRRT using ^177^Lu-DOTA-octreotate or ^90^Y–DOTA-octreotate have significant efficacy in controlling NETs that have progressed despite SSA therapy and is considered when GaTate PET uptake at tumour sites is greater than background liver uptake, indicating a sufficient target [10–14].

Administration of SSA therapy prior to GaTate PET/CT has the potential to alter radiotracer biodistribution. The EANM procedure guidelines recommend a time interval of 3–4 weeks after administration of long-acting analogues before performing GaTate PET/CT [15]. The guidelines, however, acknowledge that the effects of SSA therapy have not been well characterised. The aim of this study was to perform intra-individual comparison of radiotracer uptake on GaTate PET/CT in both physiologic and sites of metastatic disease at baseline and following LA-SSA therapy.

## Methods

### Study population

We retrospectively identified 21 (13 M; 8 F, Age 30–89) patients with histologically-proven metastatic NET who had a GaTate PET/CT at baseline whilst treatment naïve (scan 1) and a restaging scan after commencing LA-SSA (scan 2) without any other intervening therapies such as chemotherapy or PRRT. All studies were performed at the Peter MacCallum Centre between June 2010 and February 2014. Scan 2 was performed after a variable amount of time of SSA therapy (mean and median 6 months, range 2–12 months) at the discretion of the referring physician. We recommend different intervals before restaging depending on the grade of the tumour. For European Neuroendocrine Tumour Society (ENETS) Grade 2 tumours which may progress more rapidly there is a greater imperative to restage earlier (eg. 3–6 months) so that other therapies such as peptide receptor radionuclide therapy (PRRT) can be used in the event of rapid progression. For ENETS Grade 1 tumours, the likelihood of progression within such a short period is remote, and we therefore recommend anatomic restaging in 6 months and GaTate PET/CT restaging in 12 months intervals, unless clinical or biochemical assessment raises suspicion of earlier disease progression. Serum chromogranin-A levels at the time of PET scans were available for comparison in 17 of 21 patients. Chromogranin-A levels were performed within 1, 2 and 3 months of the follow-up PET scan in 82%, 15% and 3%, respectively. Patient characteristics are presented in Table 1. The study constituted a clinical audit and quality assurance activity and institutional ethics approval was therefore not required. The study was undertaken in accordance with the Helsinki Declaration of 1975, as revised in 2008.

**Table 1:** Patient Characteristics

Pt	Age	NET Type	Sites of SSTR-Positive Disease	Rx	Dose	Vol 1	Vol 2	CG1	CG2
1	40	SB Carcinoid	Nodes	12	30 S	3.22	5.1	< 1.6	< 1.6
2	61	Pancreatic	Pancreas, Lungs	8	30 S	4.3	11	51	16
3	55	SB Carcinoid	Small Bowel, Nodes, Peritoneal	6	30 S	6.1	5.1	62	32
4	53	SB Carcinoid	Liver, Peritoneum, Nodes	12	30 S	7.9	10.6	345	175
5	62	SB Carcinoid	Nodes	3	120 L	9.5	9.8	40	23
6	41	Bronchial	Lungs, Bones, Salivary Glands	6	30 S	12.5	12.8	9.3	< 6
7	52	SB Carcinoid	Breasts, Nodes, Liver, Peritoneal	4	30 S	13.6	14.6	NA	NA
8	39	SB Carcinoid	Bone, Liver	6	30 S	14.8	15.2	NA	NA
9	56	SB Carcinoid	Liver, Peritoneal, Ovary	5	30 S	19.2	19.8	35	14
10	89	SB Carcinoid	Small bowel, Nodes, Peritoneal	4	90 L	29.4	27.6	265	48
11	55	SB Carcinoid	Peritoneal, Nodes, Liver	5	30 S	51.8	46.7	99	101
12	66	SB Carcinoid	Small Bowel, Peritoneal	4	30 S	67.2	62.4	454	111
13	80	Pancreatic	Pancreas, Liver	6	30 S	1.4	100	77	243
14	49	SB Carcinoid	Liver, Bones, Peritoneum	6	40 S	3	46.4	82	212
15	30	Pancreatic	Liver	13	30 S	7.8	45	79	640
16	66	Paraganglioma	Pelvic / Peri-hepatic masses, Bone	3	20 S	38.6	75.1	14	26
17	73	SB Carcinoid	Ileum, Nodes, Liver	5	30 S	44.1	201.2	267	NA
18	65	Bronchial	Bone, Liver	10	30 S	45.2	120.5	< 121	130
19	68	SB Carcinoid	Liver, Nodes	6	30 S	86.7	472.3	144	750
20	67	SB Carcinoid	Liver, Bones	2	30 S	205.3	297.6	NA	NA
21	65	Pancreatic	Pancreas, Liver	6	20 S	338	783	129	133

Pt = Patient Number, NET = Neuroendocrine Tumour, SSTR = Somatostatin Receptor, Rx = Months SSA therapy, Dose = Monthly SSA (mg), S = Sandostatin LAR, L = Lanreotide, Vol 1 = Total body tumour volume scan 1 (mL), Vol 2 = Total body tumour volume scan 2 (mL), CG1 = Chromogranin level (μg/L) scan 1 CG2 = Chromogranin level (μg/L) scan 2. The first 12 numbered patients are those without tumour progression between the two scans and are further detailed in Table 2

### ^68^Ga-DOTA-Octreotate (GaTate) PET/CT

^68^Ga-DOTA-Octreotate was synthesized as previously described [16, 17]. For each production, 42μg of peptide was used but the product was divided and administered to several patientsdepending on patient weight, generator yield and number of patients scheduled. The administered peptide mass therefore ranged from 10-40μg. Beginning 35–88 min after intravenous injection of 85–307 MBq ^68^Ga-DOTA-Octreotate (GaTate), patients were imaged from vertex to proximal thighs on a PET/CT scanner (Discovery 690 GE Healthcare, USA or Siemens Biograph Siemens Healthcare, Germany). A low-dose CT acquisition was obtained first followed by the PET acquisition. No fasting was required. Patients were encouraged to void during the uptake phase. For patients on LA-SSA therapy, we perform GaTate PET/CT in the week prior to next LA-SSA administration, i.e. 3–4 weeks after LA-SSA administration. A longer period after LA-SSA injection before repeating GaTate PET/CT is not feasible, particularly in patients with symptoms from hormone secretion deriving symptomatic benefit. A shorter period is more likely to result in competitive effects between LA-SSA and radiotracer. Therefore, the time period just before the next administration is most pragmatic. Importantly, the uptake time of second scan in relation to LA-SSA injection was consistent throughout the cohort. 14/21 scan pairs were performed on the same PET/CT machine with both PET/CT machines calibrated and standardized for SUV measurements.

### Image analysis

A 3-D fusion workstation (MIMvista 5.0, MIMvista Corp. Cleveland, OH, USA) was used for image analysis. For quantitation at sites of physiological GaTate uptake and metastatic disease, a 3-D volume of interest (VOI) tool was used to draw VOIs around the pituitary gland, thyroid gland, parotid and sub-mandibular glands, adrenal glands, liver, spleen and metastatic deposits to measure maximum standardized uptake value (SUVmax). Splenic activity was not analysed in one patient owing to prior splenectomy. Four small VOIs were drawn over the proximal limbs and combined together to calculate the average body background. Using an automated SUV threshold of 10 to encompass all tumour with adjustment to exclude any sites of physiologic uptake such as spleen and kidney, total body tumour volume (mL) was also measured.

### Subanalysis to account for potential confounders

Distribution of GaTate is potentially confounded by a ‘tumour sink effect’ [16] whereby higher tumour volumes act as a ‘sink’ for the injected radiotracer resulting in decreased bioavailability and lower SUV measurements at other physiologic body sites. Therefore, if significant disease progression or regression occurred between scans, this could potentially result in changes of uptake at physiologic sites. To minimize this bias, a subgroup analysis was performed in patients with stable disease between the two studies as defined by < 10% change in total body tumour volume or low (< 20 ml) total body tumour volume on both scans (Patients 1–12 Table 1). An additional sub-analysis on the cohort was performed in patients with longer or shorter uptake times following radiotracer administration.

### Statistical analysis

Statistical analysis was performed using Analyse-it (Analyse-it Software Ltd., Leeds, UK). Comparisons were made using paired student’s t tests for normally distributed variables with a two-sided *p*-value of 0.05 considered statistically significant. Bland Altman analysis was performed to evaluate variability in GaTate uptake time between scans.

## Results

### Changes in physiologic distribution

**Fig. 1:**
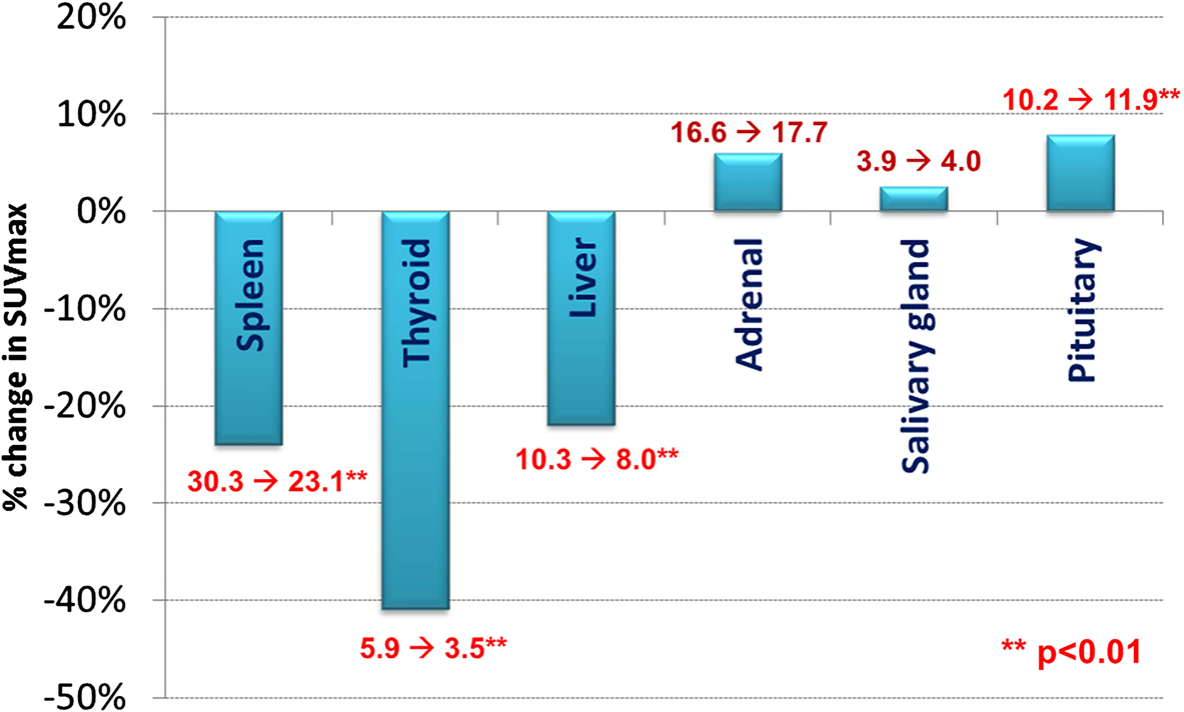
Change in mean SUVmax from baseline to repeat GaTate PET/CT after SSA therapy

**Fig. 2:**
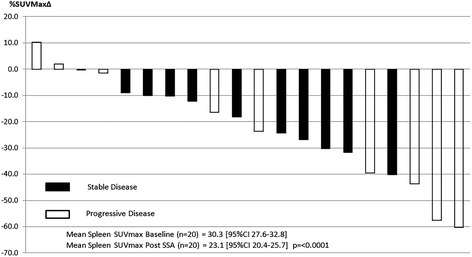
Spleen SUVMax % Δ Post SSA (*n* = 20, 1 patient prior splenectomy)

**Fig. 3:**
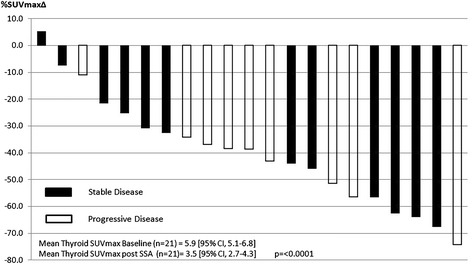
Thyroid SUVMax % Δ Post SSA (*n* = 21)

**Fig. 4:**
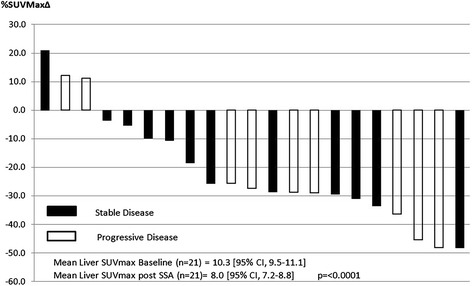
Liver SUVMax % Δ Post SSA (*n* = 21)

Splenic, thyroid and hepatic SUVmax decreased significantly for all patients (*n* = 21) between baseline GaTate PET/CT and following SSA therapy (Fig. 1). For the overall cohort, splenic SUVmax decreased by 24% from 30.3 to 23.1 (*p* = < 0.0001) (Fig. 2), thyroid by 41% from 5.9 to 3.5 (*p* < 0.0001) (Fig. 3) and liver by 22% from 10.3 to 8.0 (*p* = < 0.0001) (Fig. 4). Adrenal (*n* = 42) and salivary gland (*n* = 84, 2 glands with possible metastatic deposits excluded) SUVmax did not change significantly following SSA therapy, from 16.6 to 17.7 (*p* = 0.24) and 3.9 to 4.0 (*p* = 0.11), respectively. Pituitary gland SUVmax increased slightly following SSA therapy from a baseline of 10.2 to 11.9 (*p* = 0.004).

**Fig. 5:**
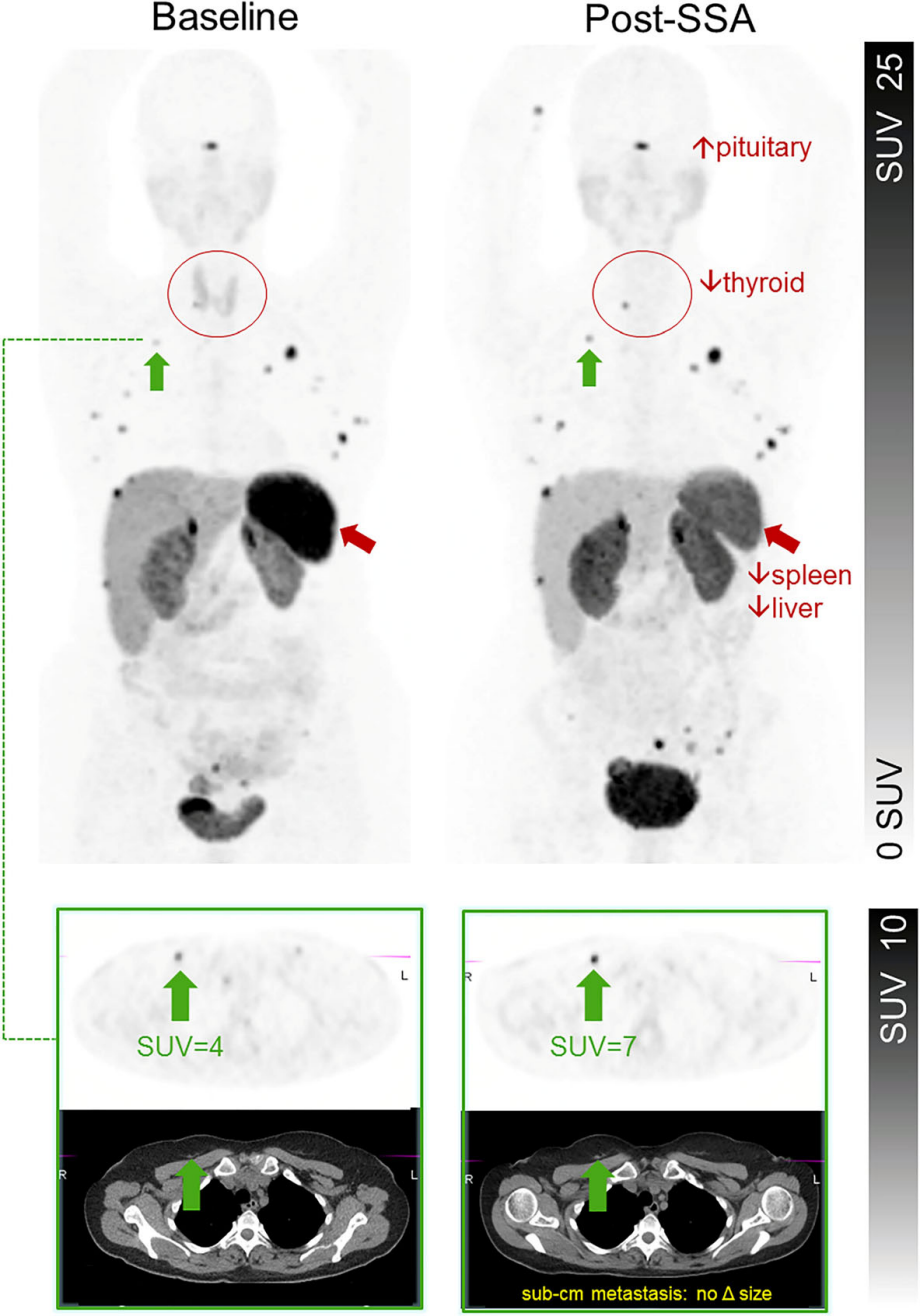
Maximum Intensity Projected Ga-68 DOTATATE PET Images of representative patient. Increase in metastatic lesion uptake post 30 mg Sandostatin LAR in the setting of decreasing serum chromogranin levels and no change in anatomic size of lesions suggests altered DOTATATE biodistribution following SSA resulting in pseudoprogression. Also note diffusely decreased thyroid uptake, which is almost universally seen in all patients following SSA

In the subgroup analysis of patients with stable disease (*n* = 12), findings were similar with mean splenic activity decreasing from 31.2 to 24.9 (*p* = 0.0006), thyroid from 5.8 to 3.4 (*p* = 0.0006) and liver from 10.4 to 8.3 (*p* = < 0.0001). No change was seen in adrenal or salivary gland SUVmax and pituitary gland SUVmax increased from 10.0 to 12.2 (*p* = 0.02).

Variability of intra-individual GaTate uptake time between scans did not appear to influence findings with significant reduction in mean splenic, thyroid and hepatic SUVmax demonstrated in patients with either longer (*n* = 12) or shorter (*n* = 9) uptake times on their second GaTate scan (Additional file 1: Figure S1, Additional file 2: Figure S2 and Additional file 3: Figure S3).

### Changes in metastatic lesion uptake

SUVmax of 49 metastatic lesions in patients with stable disease (*n* = 12) were measured at baseline and following long acting somatostatin analogue therapy (1–5 lesions measured per patient) (Table 2). 30/49 (61%) of metastatic lesions had an increase in SUVmax following SSA therapy. On a per patient analysis, metastatic lesion SUVmax increased in 7/12 (58%) patients. In 5/7 of these patients chromogranin-A levels were available for correlation and all 5 demonstrated stable or decreasing serum levels at the time of scan 2. Average metastatic lesion SUVmax decreased in 5/12 (42%) of patients with all 5 of these patients also demonstrating stable/ decreasing serum chromogranin-A levels at the time of scan 2. For bone, nodal and liver disease the SUVmax increased by 5.0 ± 11.6, 3.0 ± 10.9 and 6.2 ± 5.9, respectively.

**Table 2:** SUVmax change in 49 Metastatic Lesions in 12 patients without Progression between baseline and restaging scan

Patient	Mets	Met 1	SUVmax Δ	Met 2	SUVmax Δ	Met 3	SUVmax Δ	Met 4	SUVmax Δ	Met 5	SUVmax Δ	Av SUVmax Δ
1	1	Node	11.6	NA	NA	NA	NA	NA	NA	NA	NA	11.6
2	3	Lung	−13.9	Node	3.4	Node	−2	NA	NA	NA	NA	−4.2
3	2	Node	−13	Bone	−9	NA	NA	NA	NA	NA	NA	−11.0
4	5	Liver	8.7	Liver	2.9	Bowel	3.1	Peritoneal	9.1	Bowel	0	4.8
5	4	Bowel	−1.3	Bowel	−1.7	Node	−0.7	Bone	−1.2	NA	NA	−1.2
6	5	Bone	2.5	Bone	−4.6	Saliv	Gland	−2.5	Bone	−0.9	Bone	−8.7 -2.8
7	5	Bone	1.9	Bone	2.2	Bowel	14.1	Bone	15.2	Liver	−2.1	6.3
8	5	Liver	9.8	Liver	18.4	Liver	3.6	Liver	6.8	Bone	2.9	8.3
9	4	Bowel	5	Liver	1.6	Bone	6.5	Bone	4.5	NA	NA	4.4
10	5	Node	19.7	Bone	18	Bone	9.4	Node	16.8	Bone	37	20.2
11	5	Bowel	−7.4	Bowel	−4.7	Node	−6.2	Bowel	−10.1	Node	−2.3	−6.1
12	5	Liver	6.5	Bowel	1.4	Bowel	−1	Bowel	−1.2	Bowel	−3.2	0.5

Mets = Total number of metastases assessed per patient, Met 1–5 **=** Location of metastasis, Av SUVmax Δ = Average SUV Max Δ for all measured lesions in each patient

An analysis of metastatic lesion SUV relative to hepatic activity was also performed. In patients with stable disease, 40/49 (82%) had a SUVmax higher than liver at baseline compared to 44/49 (90%) following SSA therapy, resulting in a change in Krenning Score. Metastatic lesion:liver SUVmax ratio increased in 40/49 (82%) of lesions following SSA therapy (Table 3).

**Table 3:** Metastasis:Liver SUVmax ratio 12 Stable patients

Patient	Met 1	R1	R2	Met 2	R1	R2	Met 3	R1	R2	Met 4	R1	R2	Met 5	R1	R2
1	Node	2.72	3.51	NA	NA	NA	NA	NA	NA	NA	NA	NA	NA	NA	NA
2	Lung	3.29	1.93	Node	0.93	1.36	Node	1.44	1.30	NA	NA	NA	NA	NA	NA
3	Node	1.67	0.84	Bone	1.42	0.96	NA	NA	NA	NA	NA	NA	NA	NA	NA
4	Liver	2.62	4.04	Liver	1.57	2.12	Bowel	1.53	2.11	Peritoneal	1.43	2.77	Bowel	1.06	1.18
5	Bowel	2.39	2.76	Bowel	1.22	1.29	Node	0.90	1.01	Bone	0.76	0.78	NA	NA	NA
6	Bone	0.76	1.08	Bone	1.05	0.54	Saliv Gland	2.28	2.06	Bone	0.92	0.85	Bone	4.60	3.73
7	Bone	2.26	3.48	Bone	2.62	4.04	Bowel	1.58	4.36	Bone	1.47	4.37	Liver	2.47	3.18
8	Liver	1.73	3.83	Liver	1.61	4.89	Liver	1.38	2.44	Liver	1.18	2.63	Bone	0.72	1.43
9	Bowel	0.72	1.29	Liver	2.16	2.57	Bone	1.58	2.40	Bone	3.44	4.28	NA	NA	NA
10	Node	2.20	5.70	Bone	1.76	4.83	Bone	0.83	2.39	Node	1.51	4.32	Bone	2.20	7.80
11	Bowel	1.71	1.52	Bowel	2.64	3.05	Node	3.22	3.69	Bowel	2.78	2.68	Node	3.10	3.93
12	Liver	1.43	3.71	Bowel	0.96	2.06	Bowel	1.30	2.35	Bowel	1.05	1.84	Bowel	2.91	5.13

Met 1–5 **=** Location of metastasis, R1 = Baseline Metastasis:Liver SUVmax ratio R2 = Post SSA Metastasis:Liver Uptake ratio

## Discussion

Our findings demonstrate that long-acting SSA therapy has variable effects on physiological ^68^Ga-DOTA-Octreotate uptake in different organs with reduction of uptake in the thyroid gland, spleen and liver, slight increase in uptake in the pituitary gland and no effect on salivary and adrenal gland uptake. By performing a sub-analysis in patients with relatively stable disease between scans, we were able to confidently exclude ‘tumour sink effect’ as a contributing cause for these changes. The changes were also not explained by differences in uptake period.

The approximate 25% and 20% reduction in physiologic splenic and hepatic GaTate SUVmax demonstrated following SSA therapy has implications for both interpretation of imaging and management of patients for PRRT. Our study demonstrated SSA therapy increased metastatic lesion:liver uptake ratio in 82% of lesions, thereby potentially increasing the Krenning score, a visual scoring system which uses tumour intensity relative to liver and spleen to grade uptake [18]. Although originally developed for interpretation for planar Indium-111 octreotide scanning, we and other groups apply the same scoring for GaTate PET/CT. The increase in metastatic lesion:liver uptake ratio was primarily due to a decrease in liver SUVmax.

These results have significant implications for interpretation of GaTate PET/CT for response assessment following commencement of SSA therapy. Increased tumour to hepatic and splenic ratio may thereby result in increase in Krenning Score which may be misinterpreted as disease progression. Moreover, SUVmax of metastatic lesions increased in 58% patients with stable disease. Correlation with stable or decreasing Chromogranin-A in these patient support the rationale that the change in uptake intensity merely reflected altered biodistribution. In patients without interval disease progression in whom tumour SUVmax increased, the change did not meet the EORTC criteria of 25% increase [19] in intensity to define progressive disease. Furthermore, the increased sensitivity could result in visualization of small volume disease not seen at baseline. In our experience, this is most likely to occur with sub-cm lesions subject to partial volume effects. Our findings also suggest that SSA therapy increases the likelihood of a patient being considered suitable for PRRT, as most groups use a Krenning Score of 3 or greater (uptake greater than background liver activity) to determine suitability [10].

Our results also have significant implications for delivery of PRRT, with agents such as ^177^Lu or ^90^Y DOTA-octreotate. Of most relevance is the decrease in splenic uptake following SSA therapy, as myelosuppression secondary to bystander splenic irradiation may be a potential dose limiting factor of PRRT [20, 21]. Our findings suggest pretreatment with SSA therapy reduces physiologic splenic uptake, and therefore may reduce total splenic radiation exposure from PRRT and related myelosuppression. The higher tumour uptake potentially increasesthe therapeutic index of PRRT. These findings raise the possibility that is somewhat analogous to the administration of ‘cold’ Rituximab to saturate physiological binding sites prior to treatment with radiolabelled Rituximab in patients with B cell lymphoma to increase binding at sites of disease [22]. The administration of LA-SSA therapy prior to PRRT may improve efficacy of PRRT in a proportion of patients by altering biodistribution and increasing binding in metastatic lesions (Fig. 5). On the contrary, however, it does appear to decrease metastatic lesion uptake in a proportion of patients potentially rendering PRRT less effective in some patients.

The increase in pituitary gland uptake, decrease in thyroid uptake and lack of change in the salivary and adrenal glands following SSA therapy indicate that SSTR-uptake kinetics vary in different organs. The clinical relevance of these changes are uncertain but the authors note that absent or faint thyroid uptake on GaTate PET/CT is a feature that the patient is likely receiving SSA therapy. The significant change in splenic and liver intensity also cautions against using these in isolation as reference organs for windowing nuclear medicine images; scaling images according to a fixed SUV threshold may be preferred.

Our results are supported by Velikyan et al. [23] in which different doses of short-acting SSA administered immediately prior to injection of ^68^Ga-DOTATOC for PET scanning influenced the degree of NET uptake, with a dose of 50 μg Octreotide associated with increased NET uptake and higher doses of 250 μg and 500 μg associated with a decrease in NET uptake in the same patient. The majority of patients (16/21) in our study were receiving 30 mg Sandostatin LAR monthly, with the remainder (5/21) on 20 or 40 mg Sandostatin LAR or 90-120 mg of Lanreotide monthly that was ceased at least 4 weeks prior to the second PET scan. It is uncertain how much biologically active SSA was present in each patient at the time of the second PET scan and whether changes demonstrated were due to residual biologically active SSA or whether they were longer term effects of prior SSA exposure that was no longer active at the time of scanning.

We acknowledge there are several significant limitations of this retrospective study including variability of GaTate uptake times between scans performed in each patient and 7/21 scan pairs being performed on different PET scanners. All PET/CT cameras at our institution are calibrated and standardized for SUVmax measurements so any differences between machines would be expected to be minimal. Differences in intra-individual GaTate uptake times between scans appeared to have minimal influence on findings with significant reduction in mean splenic, thyroid and hepatic SUVmax demonstrated in patients with either longer or shorter uptake times on their second GaTate scan. A further limitation was the large variation in time (2–13 months) that had elapsed between baseline and post SSA PET scans with any tumour progression or regression occurring during this interval likely to directly affect metastatic lesion uptake. We accounted for this as best possible by only measuring changes in metastatic lesion uptake in patients with relatively stable disease between scans.

Despite the above limitations, we believe our findings are important and contribute to the paucity of literature evaluating the effects SSA therapy has on GaTate uptake at physiological and metastatic sites in NET patients and potential implications this has for PRRT. Based on these results, it is quite possible that the EANM procedure guideline recommendations of waiting 3–4 weeks after administration of long-acting somatostatin analogues before performing GaTate PET/CT may not be justified as an earlier timepoint could provide greater sensitivity in some patients [15]. It does, however, appear appropriate to perform GaTate PET/CT at a consistent timing relative to administration of long-acting somatostatin analogues. Nevertheless, given the changes that in biodistribution it appears that a consistent time point follow LA-SSA should be used for consistency. Further prospective, more controlled research of cohorts at multiple time points following differing doses and preparation of long-acting SSA would provide further insights.

## Conclusion

Long-acting SSA therapy decreases GaTate uptake in the thyroid gland, spleen and liver but in most cases increases metastatic lesion:liver uptake ratio. This has significant implications for interpretation of GaTate PET/CT as SSA therapy may thereby increase Krenning Score or other quantitative parameters resulting in apparent progression. In patients on therapy, consistent timing of GaTate PET/CT in relation to LA-SSA administration is pragmatic to minimise any bias attributable to competitive or other effects of LA-SSA at time of imaging. Caution should be made not to interpret changes in intensity of uptake alone as progression when comparing a post-therapy to baseline LA-SSA naïve patient or when the dose of LA-SSA is changed. The changes observed after LA-SSA therapy,may increase the likelihood of a patient being deemed suitable for PRRT. Our findings also suggest predosing with SSA prior to PRRT may enable higher doses to be delivered to tumour whilst decreasing dose to normal tissues in a proportion of patients, potentially reducing myelosuppression as a consequence of lower splenic irradiation.

## Additional files

Additional file 1: Figure S1. Uptake Time and Spleen SUVMax % Δ Post SSA (*n* = 20, 1 patient prior splenectomy). (TIFF 1294 kb)

Additional file 2: Figure S2. Uptake Time and Thyroid SUVMax % Δ Post SSA (*n* = 21). (TIFF 1455 kb)

Additional file 3: Figure S3. Uptake Time and Liver SUVMax Δ Post SSA (*n* = 21). (TIFF 1315 kb)

## Abbreviations

CgA: Chromogranin-A
ENETS: European Neuroendocrine Tumour Society
GaTate: ^68^Ga-DOTATATE
LA-SSA: Long-acting somatostatatin analogue
NET: Neuroendocrine tumour
PRRT: Peptide receptor radionuclide therapy
SSA: Somatostatatin analogue
SSTR: Somtatostatin receptor
SUV: Standardised uptake value
