# Supplementary figures and images for: Changes in biodistribution on 68Ga-DOTA-Octreotate PET/CT after long acting somatostatin analogue therapy in neuroendocrine tumour patients may result in pseudoprogression
Source: Cancer Imaging. 2018 Jan 24;18:3. doi: 10.1186/s40644-018-0136-x (PMC5781297; doi:10.1186/s40644-018-0136-x)

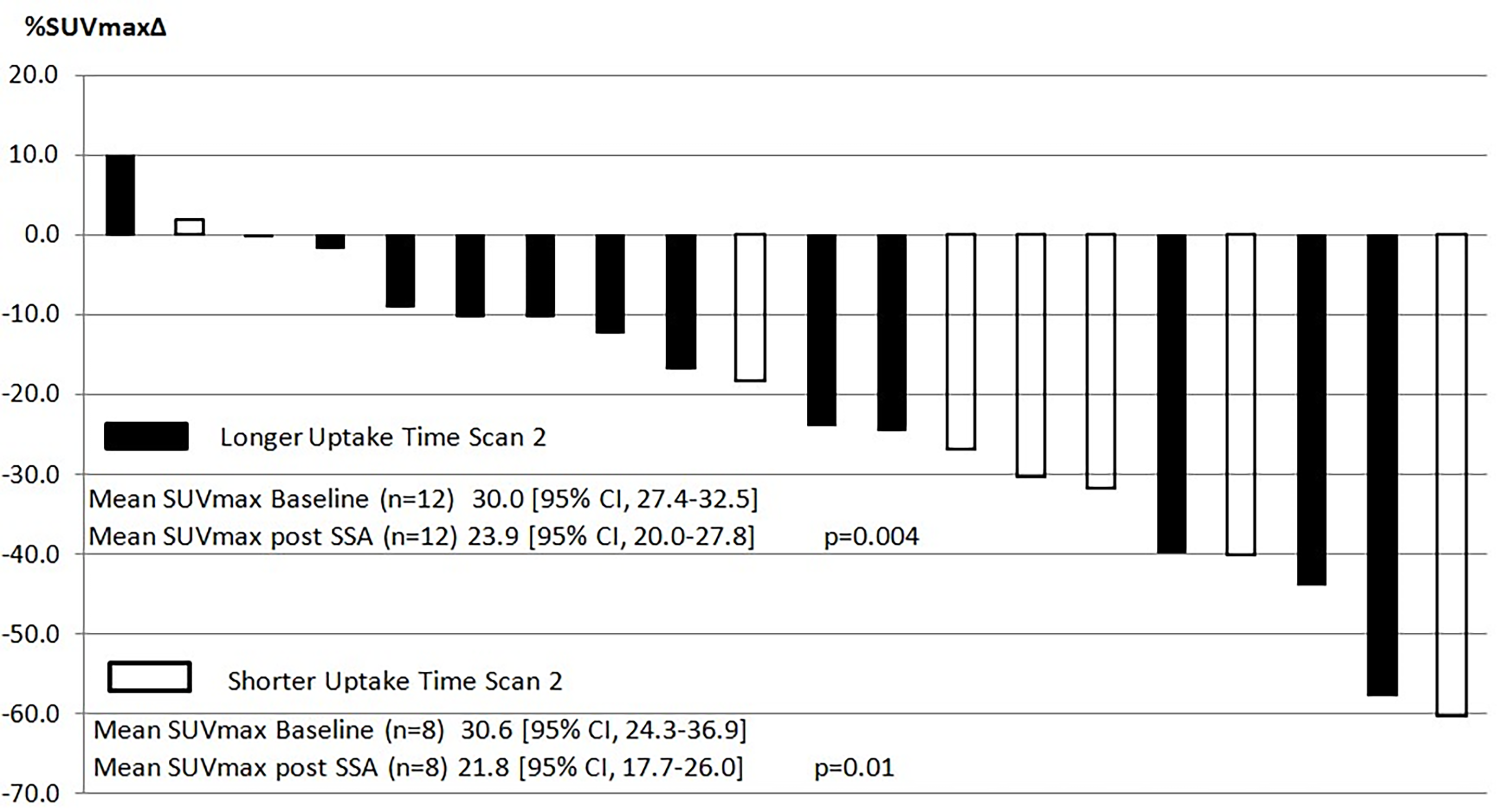

Supplement: Supplementary file 1 — Uptake Time and Spleen SUVMax % Δ Post SSA (n = 20, 1 patient prior splenectomy). (TIFF 1294 kb) [file 40644_2018_136_MOESM1_ESM.tif]

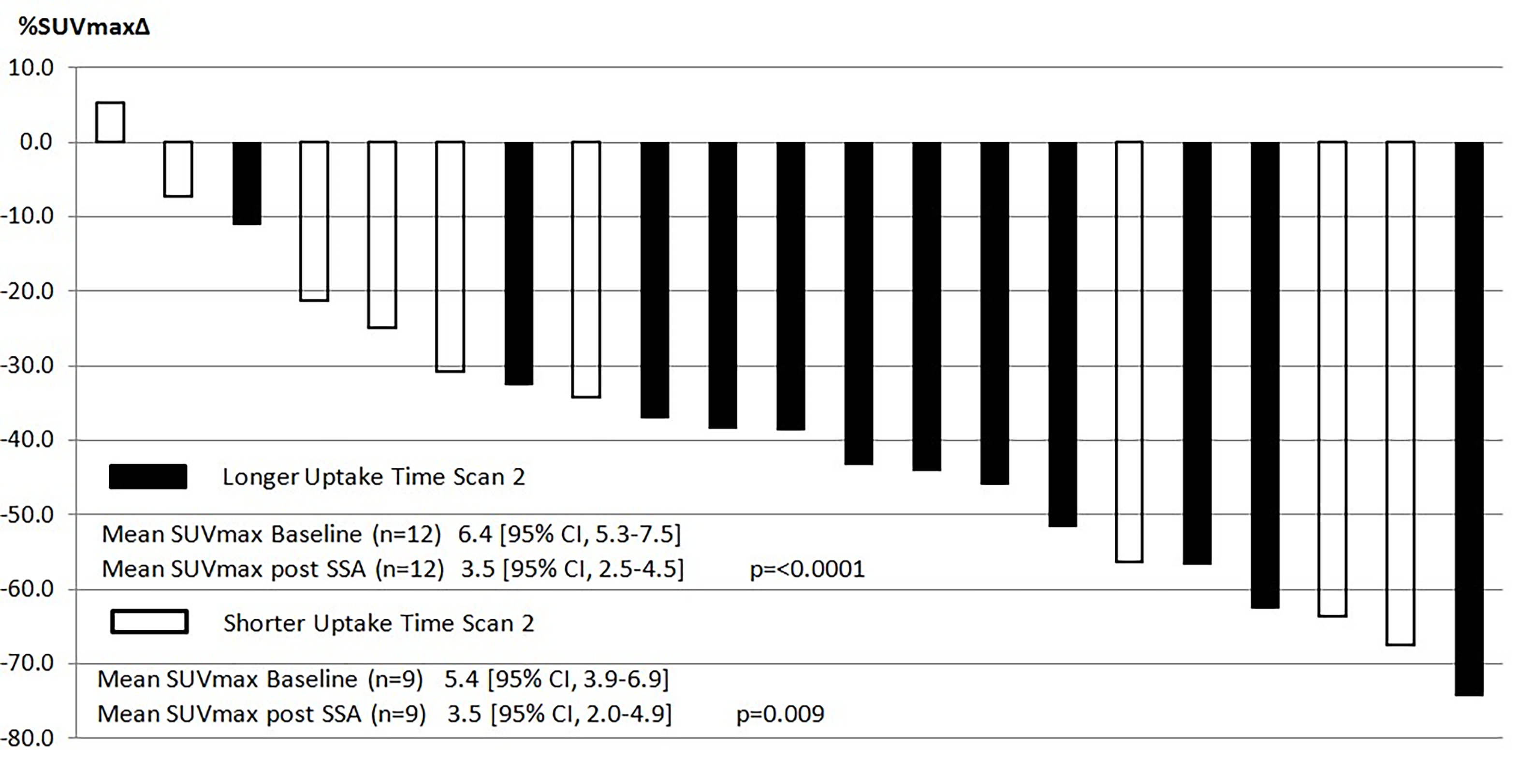

Supplement: Supplementary file 2 — Uptake Time and Thyroid SUVMax % Δ Post SSA (n = 21). (TIFF 1455 kb) [file 40644_2018_136_MOESM2_ESM.tif]

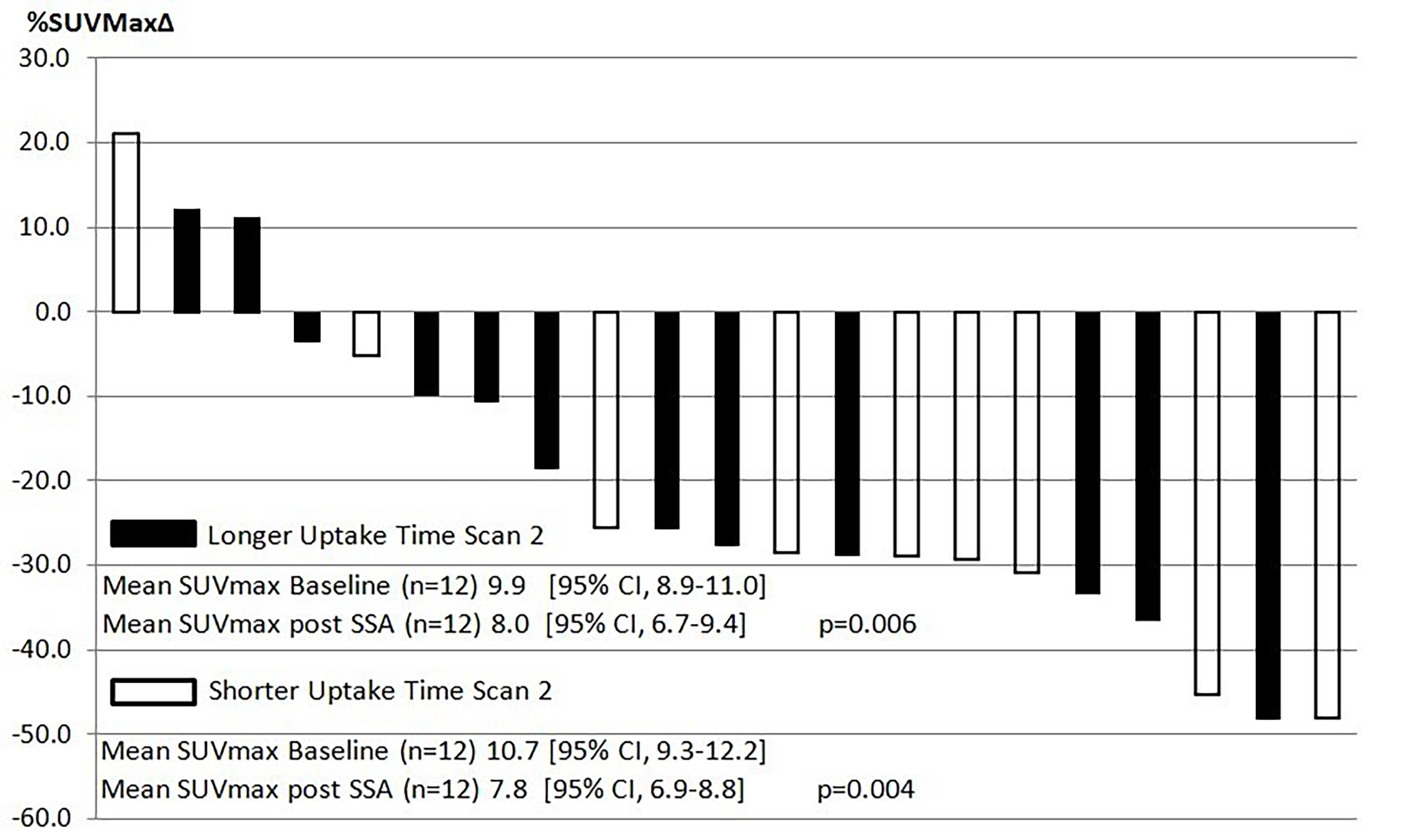

Supplement: Supplementary file 3 — Uptake Time and Liver SUVMax Δ Post SSA (n = 21). (TIFF 1315 kb) [file 40644_2018_136_MOESM3_ESM.tif]
